# Unveiling the critical roles of cellular metabolism suppression in antibiotic tolerance

**DOI:** 10.1038/s44259-024-00034-7

**Published:** 2024-06-24

**Authors:** Sayed Golam Mohiuddin, Han Ngo, Mehmet A. Orman

**Affiliations:** William Brookshire Chemical and Biomolecular Engineering Department, University of Houston, Houston, TX USA

**Keywords:** Antibiotics, Bacterial infection

## Abstract

Metabolic inhibitors are known to exhibit complex interactions with antibiotics in bacteria, potentially acting as antagonists by inducing cell dormancy and promoting cell survival. However, the specific synergistic or antagonistic effects of these inhibitors depend on factors like their mechanisms of action, concentrations, and treatment timings, which require further investigation. In our study, we systematically explored the synergistic interactions of various metabolic inhibitors—such as chloramphenicol (a translation inhibitor), rifampicin (a transcription inhibitor), arsenate (an ATP production inhibitor), and thioridazine (a PMF inhibitor)—in combination with ofloxacin. We conducted this investigation under pre-, co-, and post-treatment conditions, employing a wide concentration range and utilizing four distinct synergy models. Chloramphenicol, rifampicin, and arsenate consistently showed minimal synergy scores, indicating a notable antagonistic relationship with ofloxacin across all models and conditions. In contrast, thioridazine consistently demonstrated elevated synergy scores, especially in pre- and co-treatment scenarios, albeit its synergy decreased during post-treatment conditions. When multivariable linear regression analyses were used for all drugs and conditions examined, a correlation between the synergy of thioridazine and its ability to suppress cellular energy metabolism became evident, underscoring the potential utility of certain metabolic inhibitors as effective anti-persistence adjuvants.

## Introduction

The emergence of antimicrobial tolerance and resistance presents a critical global public health challenge, posing a substantial threat to human health. Bacterial survival mechanisms against antibiotics can be broadly classified into reversible (tolerance) and irreversible (resistance) categories. Reversible mechanisms, such as those observed in persisters, viable but nonculturable cells, and stationary-phase cells, involve non-mutational changes in bacterial growth and behavior^1^. These cells, formed in response to various stress factors, exhibit transient antibiotic tolerance, transitioning between tolerant and sensitive states^2–5^. Irreversible mechanisms, on the other hand, involve heritable changes linked to mutagenic processes, giving rise to antibiotic-resistant mutants^6^. These mutants can proliferate even in the presence of antibiotics^7^. Despite distinct meanings, there is a relationship between tolerance and resistance, as antibiotic-tolerant cells can act as reservoirs for the emergence of antibiotic-resistant mutants^8–10^. This is particularly concerning given the association of antibiotic-tolerant cells with recurrent infections, especially in biofilms where persister cells evade the immune system^11^.

The challenge of eradicating bacterial cells that are either resistant or tolerant to antibiotics is considerable, given the complex and abundant mechanisms that contribute to their formation and survival. A promising avenue for developing antibacterial therapies involves identifying a shared mechanism spanning diverse cell types, potentially found within the metabolic processes of bacterial cells. One potential target is the bacterial proton motive force (PMF), which may apply to both antibiotic-sensitive and resistant bacteria^12,13^. However, this may seem counterintuitive for tolerant cells like persisters or stationary phase cells, typically considered non-growing and dormant^14^. The conventional belief in antibiotics’ efficacy against proliferating bacteria has led to the idea that persister tolerance results from temporary growth inhibition^14^. Numerous studies supporting this hypothesis involve comparing normal cells to antibiotic-tolerant cells induced by bacteriostatic chemical treatments^15–18^. Despite persisters being in a reported non-proliferating state, they must sustain viability by maintaining a minimum adenylate energy charge, synthesizing RNA and proteins linked to repair mechanisms, and restoring antibiotic-induced damage^19,20^. Studies indicate that persister cells may consume specific metabolites^21,22^, exhibit distinct energy metabolism^23,24^, and be vulnerable to quinolone damage, necessitating energy-dependent DNA repair mechanisms for survival^25,26^. The importance of active energy metabolism for persister cell survival does not necessarily contradict prior research, as persister cells may still require energy molecule production, even if it occurs at a lower rate than in rapidly growing exponential-phase cells. Our extensive research in this field reveals that inhibitors targeting cellular energy metabolism are detrimental to persister cells^12,27^, supporting a model of altered metabolism in these cells. However, further studies are essential to determine the viability of these agents as an effective treatment strategy for persisters.

In our prior investigation, we demonstrated that heterocyclic phenothiazines (e.g., thioridazine (TDZ), chlorpromazine), commonly utilized as antipsychotic medications, effectively diminish persister levels across various bacterial strains^12,13^. This includes *Escherichia coli*, *Pseudomonas aeruginosa* (an opportunistic pathogen notorious for causing persistent lung infections in cystic fibrosis patients), highly virulent strains of *Klebsiella pneumoniae* and *Acinetobacter baumannii*, and methicillin-resistant *Staphylococcus aureus*^12,13^. Our research also demonstrated that phenothiazines can eradicate persister cells originating from quinolone antibiotics like ciprofloxacin, norfloxacin, levofloxacin, and moxifloxacin, along with beta-lactam antibiotics like ampicillin^12^. When administered at sublethal concentrations, phenothiazines impede cellular repair and recovery processes, transiently modify membrane integrity, and disrupt the proton concentration gradient across the cell membrane, ultimately disrupting the PMF^12^.

While phenothiazines have been shown to reduce cell survival fractions in the presence of antibiotics, this effect was not observed with other known metabolic inhibitors such as chloramphenicol (CAM; a translation inhibitor), rifampicin (RIF; a transcription inhibitor), and arsenate (ARS; an ATP production inhibitor)^12,15–18^. The underlying cause of this phenomenon remains unclear; however, persister cells damaged by antibiotics may rely on specific levels of transcription/translation activities and energy molecules for survival. It appears that highly potent metabolic inhibitors may prove detrimental to persisters by irreversibly impairing these crucial processes^12^. Furthermore, the impact of treatment time and concentrations of metabolic inhibitors on persister survival is yet to be fully understood and warrants further investigation. In this study, we systematically investigated the combination of various inhibitors, including CAM, RIF, ARS, and TDZ, with ofloxacin using multiple synergy models. TDZ consistently demonstrated strong synergy, particularly in pre- and co-treatment conditions, while CAM, RIF, and ARS showed minimal synergy, indicating antagonistic effects. Furthermore, multivariable linear regression analyses revealed a correlation between the efficacy of metabolic inhibitors in eradicating antibiotic-tolerant cells and their ability to suppress cellular energy metabolism. Overall, our findings underscore the importance of thorough synergistic analysis in evaluating the potential of metabolic inhibitors as anti-persistence adjuvants.

## Results

### TDZ effectively eliminates antibiotic-tolerant cells in pre- and co-treatment, with limited impact in post-treatment conditions

**Fig. 1:**
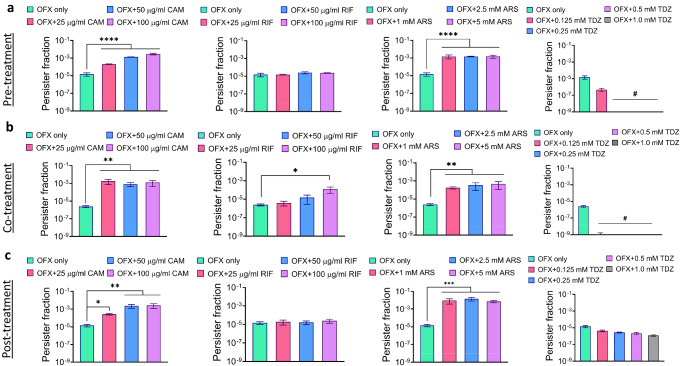
Impact of metabolic inhibitors on OFX tolerance. Cells were exposed to OFX (5 μg/ml) after 5 h of cell growth. Metabolic inhibitors were introduced at specified concentrations either 1 h before OFX addition (**a**: pre-treatment), simultaneously with OFX addition (**b**: co-treatment), or 1 h after OFX addition (**c**: post-treatment). Following a 20-h OFX treatment period, clonogenic survival assays were performed to quantify persister cells (see “Materials and methods”). In these assays, cell cultures before and after OFX treatments were washed to reduce chemical concentration, and then, serially diluted and plated on agar media for initial cell and persister quantification. Persister fractions were quantified as the ratio of persister counts to initial cell counts per ml of culture (refer to Supplementary Table 1). For pairwise comparisons between OFX only and OFX + metabolic inhibitor groups in each panel, a one-way ANOVA with Dunnett’s post-test was employed. The threshold values for statistical significance analysis were set as *P < 0.05, **P < 0.01, ***P < 0.001, and ****P < 0.0001. The mean value ± standard deviation represents the data at each time point. ♯ indicates persister levels below the limit of detection. The number of biological replicates, *N* = 3.

Metabolic inhibitors, including CAM, RIF, and ARS, have been observed to induce cellular dormancy and persistence to antibiotics across diverse microbial species^15–18^. However, phenothiazine drugs like TDZ, which inhibits cellular PMF, exhibit the remarkable ability to completely eradicate persister cells in the presence of antibiotics. Considering that the timing of treatment and concentrations of these metabolic inhibitors used as antibiotic adjuvants are crucial factors determining synergistic effects, we systematically tested the impact of these adjuvants under pre-, co-, and post-treatment conditions across a wide concentration range. Our assessment utilized a published method^12^, where cells from overnight cultures were diluted 1000-fold in fresh Lysogeny Broth (LB) medium and grown for 5 h to reach the late exponential or early stationary phase. Subsequently, cells at *t* = 5 h were subjected to ofloxacin (OFX) treatment at a concentration of 5 μg/ml for 20 h, which resulted in biphasic kill curves^12^. These experiments were carried out employing an OFX concentration that exceeded the minimum inhibitory concentration (0.039–0.078 µg/ml), enabling the quantification of antibiotic-tolerant persister cells in the cultures^7^. Notably, we selected OFX treatment due to its ability to induce DNA damage in both persister and antibiotic-sensitive cell subpopulations^25,26,28^, thereby necessitating transcriptional, translational activities, and energy molecules for repair processes. For pre-treatment conditions, metabolic inhibitors were added to the culture media at *t* = 4 h (1 h before OFX addition); for co-treatment conditions, the inhibitors were added at *t* = 5 h (simultaneously with OFX addition); and for post-treatment conditions, the inhibitors were added at *t* = 6 h (1 h after OFX addition). Additionally, it is noteworthy that, in contrast to OFX, the inhibitors at the tested concentrations exhibit bacteriostatic properties, implying that they do not independently induce bacterial cell death (Supplementary Fig. 1). Our data demonstrates that CAM, RIF, and ARS minimally affected cell survival or even enhanced persistence when co-treated with OFX in all three conditions (pre-, co-, and post-treatment, Fig. 1a–c). This aligns well with findings reported in other publications^15–18^. Conversely, TDZ exhibited a profound ability to almost eradicate antibiotic-tolerant cells in the presence of OFX under pre- and co-treatment conditions (Fig. 1a, b), with cell survival fractions falling below the limit of detection. However, this substantial impact of TDZ was not observed in the post-treatment condition (Fig. 1c).

### TDZ consistently exhibits high synergy scores, particularly in pre- and co-treatment conditions, as supported by various synergy models

To assess the existence of synergism between metabolic inhibitors and antibiotics, we calculated synergy scores. These quantitative measures are employed in drug combination studies to evaluate the combined effect of two or more drugs relative to the expected additive effect based on individual drug responses. The concept of synergy implies that the combined effect is greater than what would be predicted based on the effects of the individual drugs alone. Synergy scores provide a numerical value indicating the degree of synergy (positive score), additivity (zero score), or antagonism (negative score) in drug combinations^29^. There are several synergy models, with the most common ones being Highest Single Agent (HSA), Loewe, Bliss, and Zero Interaction Potency (ZIP). These models compare observed drug combination responses to expected responses calculated based on different assumptions about drug interactions. HSA assumes the expected response is defined by the most effective single drug^30^, Bliss takes into consideration a stochastic process in which drugs exert their effects independently^31^, ZIP considers that drugs do not potentiate each other^32^, and Loewe assumes a linear relationship between drug doses^33^ (see model equations in the “Materials and methods” section and the following references for details^29,34^). Different methods may provide consistent or divergent results. We have used all these models in our analysis, given that a consistent outcome across multiple methods increases confidence in the observed synergy.

**Fig. 2:**
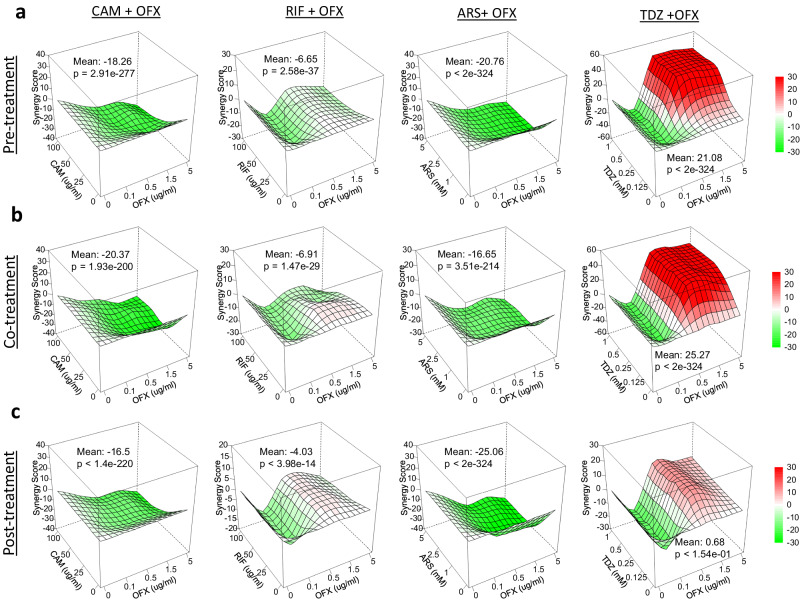
Synergy analysis, revealing higher synergy scores between TDZ and OFX. Survival fractions of cell cultures treated with combined drugs (OFX + metabolic inhibitor) or single drugs (OFX only or metabolic inhibitor only) under pre-, co-, and post-treatment conditions were utilized to determine the synergy scores^29^. **a** HSA synergy scores for all metabolic inhibitors under pre-treatment conditions (Red: higher synergy, Green: lower synergy). **b** HSA synergy scores for all metabolic inhibitors under co-treatment conditions. **c** HSA synergy scores for all metabolic inhibitors under post-treatment conditions. The mean synergy score is provided in each plot. A bootstrapping method was employed for statistical significance^29^. *N* = 3.

**Fig. 3:**
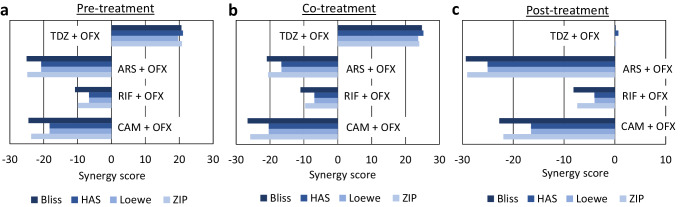
Synergy scores obtained from different models. Survival fractions of cell cultures treated with metabolic inhibitors and/or OFX under (**a**) pre-, (**b**) co-, and (**c**) post-treatment conditions were employed to calculate synergy scores using HAS, Bliss, Loewe, and ZIP models. *N* = 3.

When analyzing the sensitivity and synergy of double drug combinations using surface plots (Fig. 2), a consistent observation became evident. In Fig. 2, the mean synergy score for each condition was presented in each plot, with a color code indicating the synergy levels; red indicates strong synergy while green signifies strong antagonism. Although Fig. 2 provides HSA results, we consistently obtained similar synergy scores across all four different models for each condition (Fig. 3). This convergence enhances confidence in the reliability of our findings. CAM, ARS, and RIF always received very low synergy scores (below zero) indicating a strong antagonistic relationship (Fig. 2a–c). This pattern persisted across all models and under all conditions (pre-, co-, and post-treatments, Figs. 2a–c and 3a-c). In contrast, TDZ treatments garnered high synergy scores across all models, particularly in pre- and co-treatment conditions (Figs. 2a, b and 3a, b). However, the strength of TDZ synergy reduced during post-treatment conditions (Figs. 2c and 3c).

To methodically assess the synergy between the metabolic inhibitors and OFX, we determined synergy barometers, in which a robust synergy should be obtained when the observed response surpasses the expected responses from all four models^29^. It is important to highlight that the percent inhibition served as the chosen phenotypic response metric in these analyses, calculated as %inhibition = 100 − %viability^29^. Percent viability was determined by normalizing the logarithmic cell levels surviving the treatments to those of negative controls (untreated cultures). Therefore, a % inhibition of 100 indicates the complete sterilization of cell cultures, while a % inhibition of 0 indicates complete survival. The combination of TDZ and OFX exhibits a response exceeding 99% inhibition for both pre- and co-treatment conditions, as indicated by the pointer readout on the barometer (Supplementary Fig. 2a–c). This signifies a synergistic drug combination response, given that the expected responses from the HSA, Loewe, Bliss, and ZIP models are considerably lower, represented by the marks on the barometer. In contrast, CAM, ARS, and RIF demonstrate a response that is smaller than the expected response across all synergy models (Supplementary Fig. 2a–c), categorizing these treatments as strong antagonists. We also assessed the sensitivity of a drug combination to gauge its effectiveness. Employing a Combination Sensitivity Score (CSS) model, established in a prior study^29^, we calculated the relative inhibition of a drug combination (Supplementary Fig. 2d). This calculation is based on the area under the log10-scaled dose–response curves at the half-maximal inhibitory concentrations (IC_50_) of the individual drugs^29^. Given that a higher CSS corresponds to greater efficacy, the model highlights TDZ as a more preferable choice among the tested metabolic inhibitors for eradicating persister cells especially under pre- and co-treatment conditions (Supplementary Fig. 2d).

### The synergistic effect of TDZ arises from the correlation between its effectiveness in eliminating persister cells and its capacity to suppress energy metabolism

**Fig. 4:**
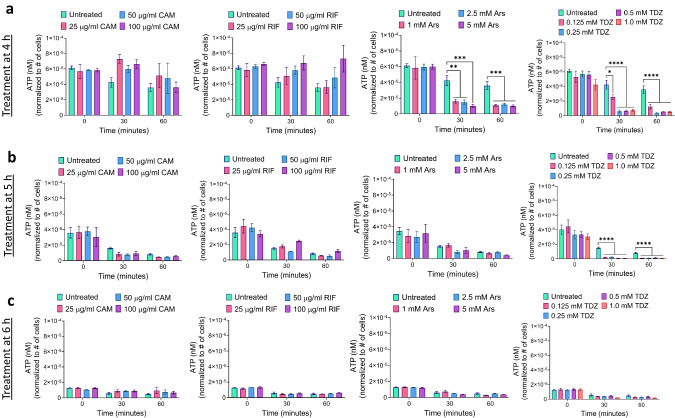
The impact of metabolic inhibitors on cellular ATP levels. Metabolic inhibitors were administered at specified concentrations at *t* = 4 h (**a**), *t* = 5 h (**b**), or *t* = 6 h of cell growth (**c**). Culture samples were collected at the indicated time points during the treatments for ATP level quantification. ATP levels were normalized to the number of cells (#) quantified by a flow cytometer. For pairwise comparisons, a one-way ANOVA with Dunnett’s post-test was applied. The threshold values for statistical significance analysis were established as *P < 0.05, **P < 0.01, ***P < 0.001, and ****P < 0.0001. The mean value ± standard deviation represents the data at each time point. *N* = 3.

The inhibitors tested in this study are commonly utilized to investigate persister mechanisms due to their impact on crucial metabolic processes, given that these chemicals either directly inhibit transcription/translation or ATP production. To explore the correlation between the synergistic effects of these chemicals and their inhibitory potencies, we assessed cellular energy levels and transcription/translation activities in cultures treated with these chemicals (excluding OFX, as its rapid killing may impede metabolic impact). ATP levels were measured both before and after 30- and 60-min treatments to gauge how rapidly and robustly these inhibitors deplete ATP molecules in cells at time points *t* = 4, 5, and 6 h during cell growth. It is important to highlight that ATP measurements for extended treatments were not performed because, with prolonged treatments, cells eventually reach the stationary phase, leading to a natural decline in ATP levels in untreated cultures. This inherent decline complicates the comparison between untreated and treated cells under longer treatment conditions. When we analyzed our experimental results, we did not observe a consistent trend in ATP levels for CAM, ARS, and RIF treatments (Fig. 4a–c). However, TDZ demonstrated a significant and rapid reduction in ATP levels across the majority of conditions, with this reduction being more notable in treatments administered at *t* = 4 and 5 h of cell growth (Fig. 4a–c). ATP depletion induced by TDZ is also evident in cultures subjected to OFX treatment (only the co-treatment condition was tested, Supplementary Fig. 3). We would like to note that we normalized the ATP levels to the number of intact cells quantified by flow cytometry. One caveat regarding the co-treatment cultures shown in Supplementary Fig. 3 is that cells may begin to die in the presence of OFX, potentially affecting intracellular ATP levels, which warrants further investigation.

**Fig. 5:**
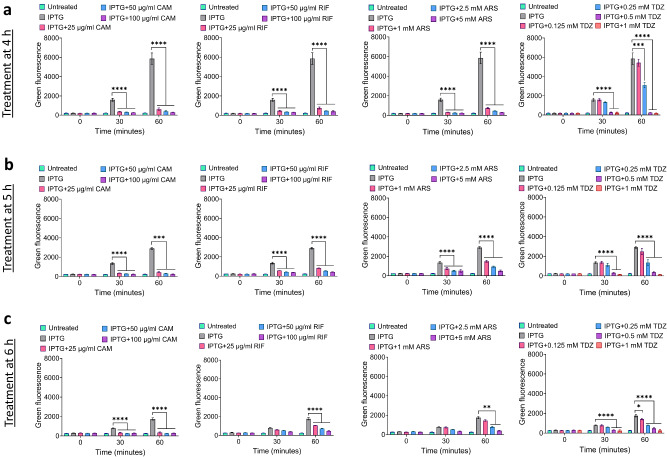
The impact of metabolic inhibitors on transcription and translation activities. Metabolic inhibitors were administered at indicated concentrations along with 1 mM IPTG (for GFP expression) at *t* = 4 h (**a**), *t* = 5 h (**b**), or *t* = 6 h of cell growth (**c**). Culture samples were collected at the indicated time points during the treatments for GFP level quantification by flow cytometry. The mean GFP values of cell populations for each biological replicate were utilized for the plots. For pairwise comparisons, a one-way ANOVA with Dunnett’s post-test was applied. The threshold values for statistical significance analysis were established as *P < 0.05, **P < 0.01, ***P < 0.001, and ****P < 0.0001. The mean value ± standard deviation represents the data at each time point. *N* = 3.

For transcription/translation measurements, we employed a reporter plasmid containing the green fluorescent protein gene (*gfp*) fused to an isopropyl β-d-1-thiogalactopyranoside (IPTG)-inducible promoter (T5), along with the *lacI*^*q*^ gene for the inhibition of the promoter in the absence of IPTG. The cells harboring this reporter were exposed to the chemicals with IPTG, and GFP measurements were conducted using flow cytometry (Supplementary Fig. 4) at the same time points as ATP measurements mentioned earlier. The rationale here is that transcription/translation inhibitors should substantially reduce GFP levels in the presence of IPTG compared to untreated cultures. While nearly all chemicals across all conditions effectively reduced GFP levels, TDZ appears to exhibit inhibitory potency at higher concentrations only (Fig. 5). A similar trend is observed in cultures subjected to OFX treatment and metabolic inhibitors (only the co-treatment condition was tested, Supplementary Fig. 5).

**Fig. 6:**
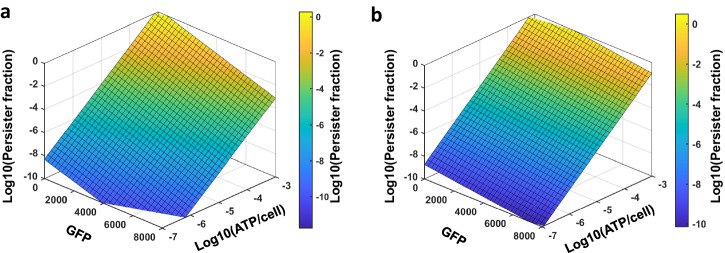
The multivariable linear regression analysis correlating the input variables to the output variables. **a** The regression analysis including all data points for metabolic inhibitors and all conditions tested (data from Figs. 1, 4, and 5). **b** The regression analysis including data from co-treatment conditions involving both metabolic inhibitors and OFX (derived from Fig. 1b and Supplementary Figs. 3 and 5). ATP and GFP measurements at 60-min time points following treatments were utilized for both analyses. The input variables (ATP and GFP levels) exhibit a significant correlation with the output variables (persister fractions) in both analyses (*P* < 0.0001, *F*-Statistics).

Although TDZ demonstrates high effectiveness in significantly reducing metabolism at higher concentrations under both pre- and co-treatment conditions, a consistent trend between GFP & ATP levels and persistence is not as evident with the metabolic inhibitors in general. It remains uncertain whether there is any correlation between the efficacy of these inhibitors in eradicating persister cells and their ability to inhibit metabolism. Assuming the GFP and ATP levels as the two input variables and persistence as the response variable, a multivariable linear regression model equation can be formulated to statistically assess the relationship between the input and response variables (${P}_{P}={\beta }_{0}+{\beta }_{1}\times {P}_{A}+{\beta }_{2}\times {P}_{R}$ where *P*_*P*_ is the log-transformed value of the persister fraction, *P*_*A*_ is the log-transformed value of the ATP level, *P*_*R*_ is the GFP level, *β*_0_ is the model intercept, *β*_1_ is the model coefficient of the ATP level, and *β*_2_ is the model coefficient of the GFP level). When analyzing the data of ATP and GFP expression levels, along with cell survival fractions across all inhibitor concentrations and treatment conditions (data from Figs. 1, 4, and 5), we identified a robust and statistically significant correlation between input and response variables (*P* < 0.0001, *F*-Statistics; Fig. 6a). The surface plot distinctly illustrates a clear correlation between persistence and ATP levels, implying that potent metabolic inhibitors capable of drastically reducing ATP levels might also eradicate persister cells, although persistence seems to be less dependent on GFP levels (Fig. 6a). This trend is particularly evident in the experimental data of ATP measurements following TDZ treatments, where a more pronounced impact on ATP levels is observed, especially at the 4 and 5-h treatments, resulting in the complete eradication of persister cells (Figs. 1a, b and 4a, b).

For our multivariable analysis presented in Fig. 6a, we utilized ATP and GFP data derived from cultures treated with metabolic inhibitors in the absence of OFX (see Figs. 4 and 5). Upon performing this analysis for data from co-treatment conditions involving both metabolic inhibitors and OFX (refer to Supplementary Figs. 3 and 5), we still observed a consistent and statistically significant correlation between input and response variables (*P* < 0.0001, F-Statistics) (Fig. 6b). Despite slight discrepancies between the outcomes illustrated in Fig. 6b compared to Fig. 6a, attributable to the absence of pre- and post-treatment conditions involving both metabolic inhibitors and OFX, our findings indicate that potent metabolic inhibitors, which markedly reduce ATP levels, may exhibit promising efficacy in eliminating persister cells within co-treatment cultures.

## Discussion

In this study, the effectiveness of metabolic inhibitors, including TDZ, CAM, RIF, and ARS, in targeting antibiotic-tolerant persister cells was investigated under pre-, co-, and post-treatment conditions. TDZ displayed a remarkable ability to almost eradicate antibiotic-tolerant cells, particularly in pre- and co-treatment scenarios, while CAM, ARS, and RIF exhibited limited impact across all conditions. This finding aligns with prior research indicating that CAM, ARS, and RIF have the potential to enhance persistence in diverse microorganisms, contingent upon treatment conditions^12,15–18,35–37^. Thorough synergy analysis using multiple models consistently highlighted TDZ’s high synergy scores, especially in pre- and co-treatment conditions, suggesting its potential as an effective antibiotic adjuvant. Multivariable linear regression analysis further suggested that the synergy of TDZ with OFX may stem from its ability to reduce energy levels to subthreshold levels crucial for persister cell survival.

In our findings, it was observed that while ARS treatment reduced ATP levels under pre-treatment conditions, its impact on energy was less evident compared to PMF inhibitors. ARS displays similarities to phosphate across a range of biologically relevant conditions^38,39^. The physicochemical resemblance between phosphate and ARS plays a role in the biological toxicity of ARS^40^, as metabolic pathways designed for the phosphate group cannot differentiate between the two molecules^38,39^. There is a potential for ARS to compete with phosphate, but this competition may not lead to a significant ATP depletion, unlike the notable depletion observed in cells treated with TDZ.

Identifying synergistic drug combinations is crucial for achieving enhanced therapeutic efficacy, minimizing side effects by potentially reducing individual drug doses, and addressing issues such as antibiotic resistance^41–43^. By quantifying the interaction between drugs, synergy scores contribute to more informed decision-making in drug development, personalized medicine, and clinical treatment strategies^29,44,45^. To prioritize potential drug combinations, it is necessary to identify those with higher CSS and higher synergy scores since CSS indicates the efficacy of a drug combination, and the synergy score indicates the degree of interaction^29^. Our study unveils that the PMF inhibitor (TDZ) demonstrates increased potency with significant synergy and CSS scores while capable of rapidly and substantially reducing cellular energy levels. PMF is a key factor in bacterial processes, consisting of two components: electric potential (ΔΨ) and transmembrane proton gradient (ΔpH). Its stability is vital for various bacterial activities, such as nutrient transport, toxin efflux, flagella motility, and pH homeostasis^46^. Compounds that disrupt PMF have the potential to combat bacterial infections, as demonstrated by certain FDA-approved drugs like daptomycin and telavancin, which disrupt the ΔΨ component^47,48^. Moreover, synthetic compounds such as JBC 1847 and oritavancin, along with antimicrobial peptides like WRK-12 and WW307, have shown antibacterial activities by disrupting ΔΨ through membrane depolarization^49–52^. Natural products like flavones and synthetic compounds like halicin, on the other hand, have been found to dissipate ΔpH^53,54^. Additionally, disrupting PMF is proposed as a strategy to prevent the transmission of antibiotic resistance genes through plasmid-mediated horizontal gene transfer^46^. This approach offers a potential avenue to mitigate the global crisis of antimicrobial resistance.

Through screening a small chemical library in our previous study, we identified a specific subset of compounds (including nordihydroguaiaretic acid, gossypol, trifluoperazine, and amitriptyline) that effectively disrupted PMF in methicillin-resistant *S. aureus* (MRSA) cells by either dissipating the transmembrane electric potential or the proton gradient^13^. We observed that known PMF inhibitors like carbonyl cyanide 3-chlorophenylhydrazone or polymyxin-B were not as effective as newly discovered PMF inhibitors or TDZ. This was attributed to the efficacy of the PMF inhibitors in eradicating MRSA cells being directly linked to their capability to disrupt PMF and permeabilize membranes^13^. PMF inhibitors may present significant challenges to persister cells by permeabilizing their membranes, depleting their energy molecules, and impairing their transcription and translation activities^12^. Despite persister cells exhibiting reduced cellular activities, they may still remain susceptible to antibiotic damage^25,28,55,56^, and consequently, may need transcription/translation activities for synthesizing repair proteins^25,26^ as well as energy molecules for energy-dependent repair mechanisms^12^. Theoretically, certain metabolic inhibitors could eradicate persister cells by obstructing these critical survival processes, while others might induce dormancy and enhance survival. However, the efficacy of these drugs may vary depending on their mechanisms of action, treatment duration, and concentration levels. For instance, the OFX-induced SOS response not only triggers the activation of DNA repair mechanisms but also inhibits cell growth and PMF through SulA and TisB proteins^57,58^; this inhibition may also reduce the interactions between the metabolic inhibitors and their targets during post-treatment. Overall, while our study highlights the dual nature of metabolic inhibitors, our findings offer valuable insights into the synergistic potential of certain inhibitors in combating antibiotic-tolerant cells, paving the way for future developments in antipersister strategies.

## Materials and methods

### Bacterial strains, chemicals, media, and culture conditions

The wild-type strain utilized in this study was *Escherichia coli* K-12 MG1655. A previously generated pUA66 plasmid, expressing a green fluorescent protein gene under the regulation of T5 promoter and *LacI*^*q*^ was used to measure cellular transcription and translation activities^12^. Chemicals were procured from Fisher Scientific (Atlanta, GA), VWR International (Pittsburgh, PA), or Sigma Aldrich (St. Louis, MO), unless otherwise specified. Ofloxacin (OFX) for persister assays, kanamycin for plasmid retention, and IPTG for GFP expression were prepared following established protocols^12^. In our previous study, the minimum inhibitory concentration (MIC) of ofloxacin was determined to be within 0.039–0.078 μg/ml^27^. TDZ (0.1 M) and sodium hydrogen arsenate heptahydrate (ARS; 1 M) stock solutions were prepared in DI water, while the CAM (50 mg/ml) solution was made in 100% ethanol. RIF (50 mg/ml) stock solutions were prepared in dimethyl sulfoxide (DMSO). All chemical solutions, except those in DMSO, underwent sterilization using 0.2 μm syringe filters. LB liquid and agar were prepared following standard procedures^12^. Overnight pre-cultures prepared from a 25% glycerol stock culture stored at −80 °C and were grown for 24 h at 37 °C with shaking at 250 rpm in 14-ml round-bottom Falcon test tubes containing 2 ml LB. Experimental cell cultures, referred to as main cultures, were prepared by diluting overnight pre-cultures (1:1000) into 25 ml fresh LB medium in 250-ml baffled flasks and cultured for 4, 5 or 6 h before treatments. Immediately prior to treatments, 2 ml of cultures from the flasks were transferred to 14-ml round bottom Falcon tubes and then subjected to treatment with metabolic inhibitors and/or OFX as outlined below.

### Chemical treatments and persister assays

The main cultures in test tubes underwent OFX (0, 0.1, 0.5, 1.5 or 5 µg/ml) treatment during the late exponential growth phase (*t* = 5 h). Chemical inhibitors—TDZ (0, 0.125, 0.25, 0.5, or 1.0 mM), ARS (0, 1, 2.5, and 5 mM), CAM (0, 25, 50, or 100 µg/ml), or RIF (0, 25, 50, or 100 µg/ml)—were introduced 1 h before OFX treatment at *t* = 4 h (pre-treatment), simultaneously with OFX at *t* = 5 h (co-treatment), or 1 h after OFX treatment at *t* = 6 h (post-treatment). Following a 20-h OFX exposure, clonogenic survival assays were performed to quantify persister cells, as detailed previously^12^. Briefly, 1 ml samples from each test tube were transferred to microcentrifuge tubes and centrifuged at 13,300 rpm (17,000 × *g*). After centrifugation, the supernatant (950 µl) was discarded, and 950 µl of phosphate-buffered saline (PBS) was added and mixed. The resulting cell suspension underwent two additional rounds of centrifugation; this washing procedure is essential to reduce the antibiotic concentration below the MIC. After the washing steps, the cells were resuspended in 100 µl PBS. For cell counting, a 10-µl sample of this suspension underwent serial dilution in a round-bottom 96-well plate, and 10 µl of the diluted cells were plated on an LB agar plate for colony-forming unit (CFU) counting. Additionally, the remaining 90 µl of undiluted cell suspension was plated to enhance the limit of CFU detection. LB agar plates were then incubated for at least 16 h at 37 °C. The CFU counts following antibiotic treatment provide persister levels, indicating the number of cells surviving the antibiotic treatment and capable of colonizing fresh medium upon removal of antibiotics. The initial cell count was determined before antibiotic treatment using the same method, and persister fractions were quantified as the ratio of persister counts to initial cell counts per 1.0 ml of assay culture. Data from cell cultures treated exclusively with OFX or a metabolic inhibitor were utilized for synergy analysis.

### ATP measurement

We used the BacTiter-Glo™ Microbial Cell Viability Assay kit (Catalog# G8230, Promega Corporation, Madison, WI, USA) to assess ATP concentrations. Following the treatment of cells with metabolic inhibitors at 4, 5 or 6 h of cell growth, 100 µl of cultures were collected at 0, 30-min, and 60-min time points. These samples were then combined with 100 µl of luciferase solutions and incubated at room temperature for 5 min. Luminescence was measured using a plate reader, with LB medium serving as a control for background luminescence. Standard curves were generated using rATP (Promega Corporation, Catalog# P1132) dissolved in LB.

### Fluorescent protein expression assay and cell counts by flow cytometry

Cells carrying the GFP reporter were cultured as described above, with the addition of 50 µg/ml kanamycin for plasmid retention. Following treatment with 1 mM IPTG and/or metabolic inhibitors at 4, 5, or 6 h of cell growth, 10 µl of cultures were collected at 0, 30-min, and 60-min time points and transferred to 990 µl PBS in flow cytometry tubes (5 ml round-bottom Falcon tubes, size: 12 × 75 mm) for GFP measurement by a flow cytometer (NovoCyte Flow Cytometer, NovoCyte 3000RYB, ACEA Biosciences Inc., San Diego, CA). For green fluorescent detection, excitation at 488 nm and a 530/30 nm bandpass filter were applied. A slow flow rate of 14 μl/min with a sample stream core diameter of 7.7 μm was used. Cells with or without GFP served to gate GFP-positive cells on flow diagrams. PBS samples with and without cells were utilized to distinguish cell populations from instrumental noise using forward and side scatter plots. Data were analyzed using Flowjo V10.10.0.

The flow cytometer in our lab can measure the number of cells and the volume of the sample analyzed. As necessary, the total cell count for the indicated cultures was determined using these measurements along with the dilution rates of the actual cell cultures.

### Synergy analysis

#### Data normalization

For synergy analysis, persister data corresponding to pre-, co-, and post-treatment conditions for each drug combination (OFX + CAM, OFX + RIF, OFX + ARS, and OFX + TDZ) presented in Fig. 1 (see data in Supplementary Table 1) were utilized. These datasets represent the combined drug response. Additionally, we incorporated single-drug response data, specifically OFX-only or inhibitor-only datasets (see Supplementary Fig. 1). Single-drug response data is essential for synergy analysis, as synergy can only be discerned by comparing the deviation between single-drug and combined-drug responses. The single-drug response data for OFX was generated by treating cells with OFX at various concentrations at *t* = 5 h (Supplementary Fig. 1), serving as the basis for all drug combinations, given that cells were consistently treated with OFX at *t* = 5 h across all conditions. Similarly, single-drug response data for metabolic inhibitors was obtained by treating cells with the inhibitors at various concentrations at *t* = 5 h (Supplementary Fig. 1). These datasets were uniformly applied across all drug combinations, as the treatment with these bacteriostatic inhibitors does not independently impact cell survival. For synergy analysis, data normalization becomes essential, as we utilized percent inhibition as a phenotypic response metric^29^:

1 $$\%\, {{\rm{Inhibition}}}=100- \%\, {{\rm{Viability}}}$$

Percent viability was calculated by normalizing the logarithmic cell levels surviving the treatments to those of negative controls that did not receive any treatment:

2 $$\% \,{{\rm{Viability}}}=\frac{{\log }_{10}({{\rm{the}}\; {\rm{CFU}}\; {\rm{level}}\; {\rm{of}}}\;{{\rm{a}}}\;{{\rm{treatment}}\; {\rm{group}}})}{{\log }_{10}({{\rm{the}}\; {\rm{CFU}}\; {\rm{level}}\; {\rm{of}}\; {\rm{the}}\; {\rm{control}}\; {\rm{group}}})}\times 100$$

#### Synergy models

When measuring the response of a drug as a % inhibition ranging from 0 to 100 (or normalized to 0 to 1), a higher value indicates better efficacy. In a combination involving *n* drugs, the observed combination response is denoted as *y*_*c*_ (ranging from 0 to 1), while the observed monotherapy response of its constituent drugs is represented by *y*_*i*_ (ranging from 0 to 1), where *i* = 1,…, *n*. Using SynergyFinder Plus tools^29^, four prominent reference models were employed to quantify the degree of combination synergy or antagonism by comparing the observed drug combination response to an expected response. These models operate under different assumptions and provide insights into the interactive effects of multiple drugs:

- The HSA model assumes that the expected response is the maximum of the single drug responses at corresponding concentrations^30^. It quantifies synergy (*S*_HSA_) by calculating the difference between the combination effect and the maximum response among the single drugs:
  3 $${S}_{{{\rm{HSA}}}}={y}_{c}-\max \left({y}_{1},\ldots ,{y}_{n}\right)$$
- The Bliss model assumes a stochastic process in which two drugs exert their effects independently^31^. The expected response is determined based on the probability of independent events^29^. The Bliss model quantifies the synergy score (*S*_BLISS_) as follows:
  4 $${S}_{{{\rm{BLISS}}}}={y}_{c}-\left(1-\mathop{\prod }\limits_{i}^{n}(1-{y}_{i})\right)$$
  where (1 − *y*_*i*_) represents the probability of drug *i* not inhibiting the target; multiplying these expressions denotes the probability that none of the drugs inhibit the target. The Bliss effect is calculated by subtracting this multiplication term from 1, providing the probability of at least one drug inhibiting the target.
- The Loewe additivity model estimates the expected response (*y*_Loewe_) as if a drug is combined with itself^29^, taking into account the individual dose–response curves of each drug and assuming a linear relationship between their doses^33^. The Loewe synergy score (*S*_Loewe_) is computed using the following equations:
  5 $${S}_{{{\rm{Loewe}}}}={y}_{c}-{y}_{{{\rm{Loewe}}}},\;{{\rm{subject}}\; {\rm{to}}}\mathop{\sum }\limits_{i}^{n}\left(\frac{{x}_{i}}{{f}_{i}^{-1}\left({y}_{{{\rm{Loewe}}}}\right)}\right)=1$$
  where *x*_*i*_ is the concentration of the drug *i*, and ${f}_{i}^{-1}\left({y}_{{{\rm{Loewe}}}}\right)$ is the inverse function of the dose response curve^29^.
- The ZIP calculates the expected response of two drugs under the assumption that they do not potentiate each other^32^. It captures drug interaction relationships by comparing changes in the potency of dose–response curves between individual drugs and their combinations:

6 $${S}_{{{\rm{ZIP}}}}=\frac{1}{n}\mathop{\sum }\limits_{i}^{n}{f}_{i}^{{\prime} }\left({x}_{i}\right)-\left(1-\mathop{\prod }\limits_{i}^{n}\left(1-{f}_{i}\left({x}_{i}\right)\right)\right)$$

where ${f}_{i}^{{\prime} }\left({x}_{i}\right)$ represents the log-logistic model defined for the combination response at dose *x*_*i*_ of drug *i* in the presence of other drugs, while ${f}_{i}\left({x}_{i}\right)$ indicates the dose response of the monotherapy predicted by a monotonically increasing curve-fitting model. Further detailed explanations regarding these models can be found elsewhere^29,34^.

#### Combination Sensitivity Score (CSS)

CSS is determined by utilizing the relative IC_50_ values of compounds and the area under their dose–response curves (AUC) as described previously^29^. The CSS for a drug combination is computed by employing a fixed concentration for one compound (background drug, e.g., OFX) and varying concentrations for another (foreground drug, e.g., a metabolic inhibitor). This process yields two CSS values, which are subsequently averaged. The dose–response for each drug is modeled using a four-parameter log-logistic curve, and the area under the log-scaled dose–response curve (AUC) is calculated using the following equation^29^:

7 $${{\rm{AUC}}}={\int_{{c}_{1}}}^{{c}_{2}}\left({y}_{\min }+\frac{{y}_{\max }-{y}_{\min }}{1+{10}^{\lambda \left({\log }_{10}{{{\rm{IC}}}}_{50}-x{\prime} \right)}}\right){\rm{d}}{x}^{{\prime} }\;{{\rm{where}}}\;{x}^{{\prime} }={\log }_{10}x$$

In the equation, *y*_min_ and *y*_max_ denote the minimal and maximal inhibition, respectively; *λ* is the shape parameter indicating the sigmoidicity or slope of the dose–response curve, and [*c*_1_*, c*_2_] represent the concentration range being tested for the foreground drug. IC_50_ is a quantitative measure indicating the amount of a specific metabolic inhibitor required to reduce cell viability by 50% in the presence of OFX.

#### Statistical significance and surface and barometer plots

Statistical significance was determined either at a single dose or the entire dose matrix level, using a bootstrapping method as described in a previous study^29^. For a two-drug combination experiment, a bootstrapping approach calculates confidence intervals for synergy scores by sampling responses from a normal distribution (see details in ref. ^29^). Synergy scores and statistical evaluations were depicted in three-dimensional and barometer plots using SynergyFinder Plus^29^.

### Multivariable linear regression analysis

We conducted a multivariable linear regression analysis to explore correlations between the response (persister levels) and input variables (ATP and GFP expression levels). ATP and GFP data at 60-min time points were utilized for this analysis. Multiple linear regression analysis was performed using GraphPad Prism 9.3.0. The linear model equation is represented as follows:

8 $${P}_{P}={\beta }_{0}+{\beta }_{1}\times {P}_{A}+{\beta }_{2}\times {P}_{R}$$

where *P*_*P*_ is the log-transformed value of the persister fraction, *P*_*A*_ is the log-transformed value of the ATP level, *P*_*R*_ is the GFP expression level, *β*_0_ is the model intercept, *β*_1_ is the model coefficient of the ATP level, and *β*_2_ is the model coefficient of the GFP level. The *F*-statistic was employed to compare the goodness of fit between the model equation, which includes parameter estimates, and a null model that lacks these estimates. The parameters identified from the regression analysis were used to generate three-dimensional plots with MATLAB.

### Statistical analysis

Three independent biological replicates were conducted for all experiments. Statistical analyses for synergy scores and the multivariable linear regression model were explained in the previous sections. For synergy analysis, we utilized SynergyFinder Plus—an interactive tool designed for analyzing drug combination dose–response data^29^. This tool facilitates efficient implementations of popular synergy scoring models such as HSA, Loewe, Bliss, and ZIP, allowing for the quantification of the degree of drug synergy^29^. For pairwise comparisons, one-way ANOVA with Dunnett’s post-test was employed. The threshold values for statistical significance analysis were set as *P < 0.05, **P < 0.01, ***P < 0.001, and ****P < 0.0001. Statistical analyses for pairwise comparisons and figure generations were performed using GraphPad Prism. The mean value ± standard deviation represents the data at each time point.

### Reporting summary

Further information on research design is available in the Nature Research Reporting Summary linked to this article.

## Supplementary information

Supplementary Information

Reporting Summary

## Data Availability

All data in this manuscript is available in either the main text or the Supplementary Information file. Additionally, raw data will be promptly provided upon request.
