# Supplementary material for: Unveiling the critical roles of cellular metabolism suppression in antibiotic tolerance
Source: NPJ Antimicrob Resist. 2024 Jun 24;2:17. doi: 10.1038/s44259-024-00034-7 (PMC11721439; doi:10.1038/s44259-024-00034-7)
Supplement: Supplementary file 1 — Supplementary Information [file 44259_2024_34_MOESM1_ESM.pdf]

UNVEILING THE CRITICAL ROLES OF CELLULAR METABOLISM SUPPRESSION IN ANTIBIOTIC TOLERANCE

Sayed Golam Mohiuddin, Han Ngo, Mehmet Orman*
William Brookshire Chemical and Biomolecular Engineering Department, University of Houston, Houston, Texas, USA
Corresponding author: morman@central.uh.edu

SUPPLEMENTARY INFORMATION

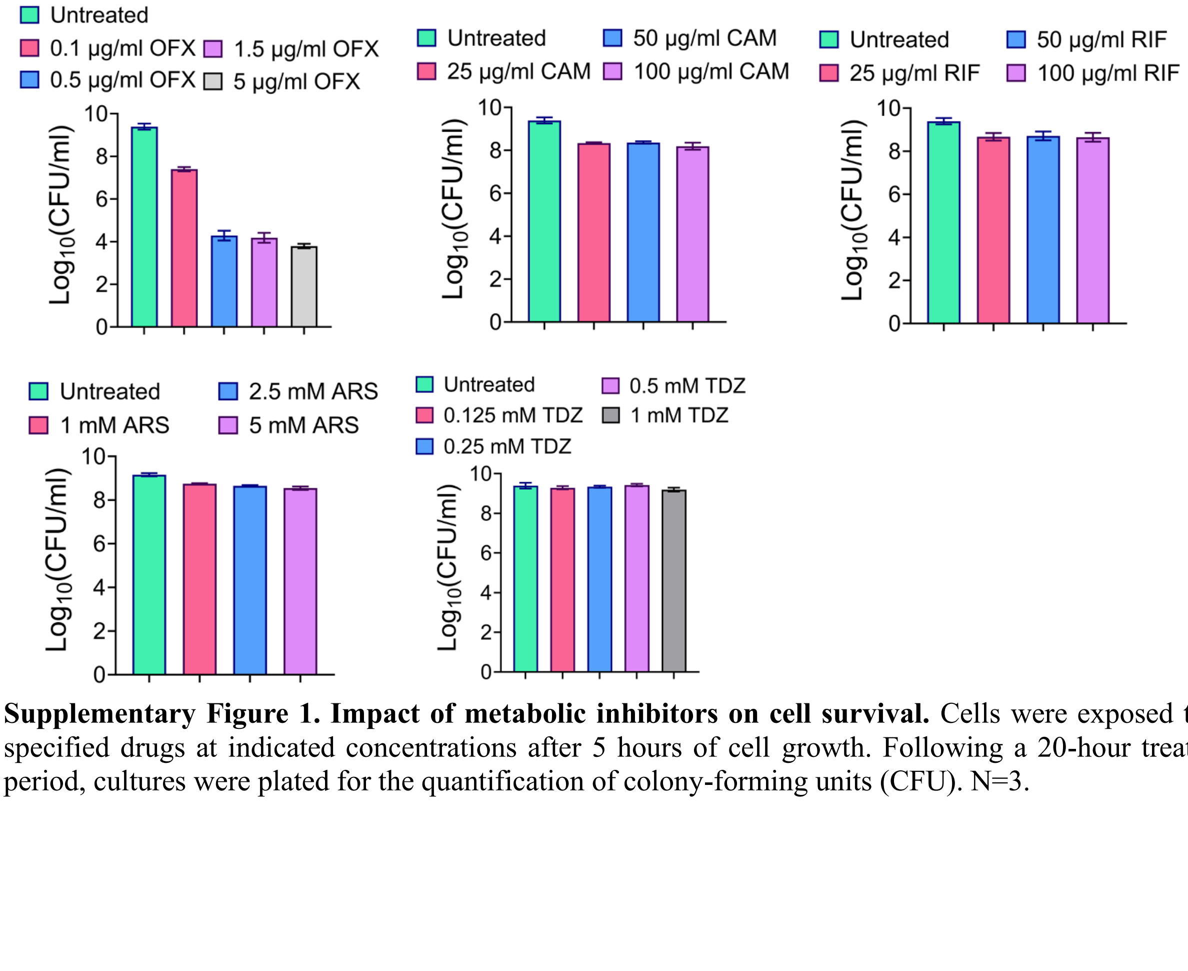

Supplementary Figure 1. Impact of metabolic inhibitors on cell survival. Cells were exposed to the specified drugs at indicated concentrations after 5 hours of cell growth. Following a 20-hour treatment period, cultures were plated for the quantification of colony-forming units (CFU). N=3.

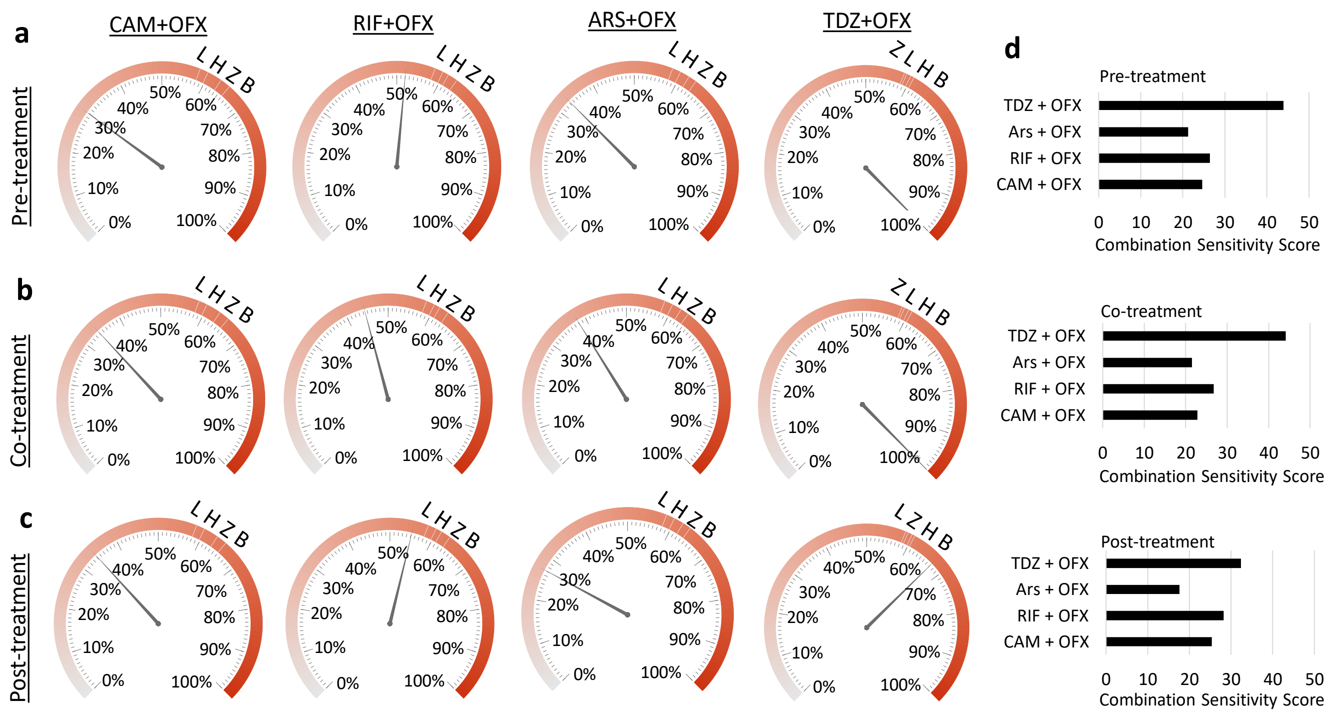

Supplementary Figure 2. Synergy analysis, demonstrating an increased level of robust and effective synergism between TDZ and OFX. Panels a-c feature barometers with pointer readouts, showing the observed inhibition response for metabolic inhibitors in (A) pre-, (B) co-, and (C) post-treatment conditions. Robust synergy is indicated when the observed response exceeds the expected response, while robust antagonism is observed when the observed response is smaller than the expected response. The expected responses of the synergy models, represented by L (Loewe), H (HSA), Z (ZIP), and B (Bliss) letters, are highlighted in each barometer. In **Panel d**, combination sensitivity scores for metabolic inhibitors are presented under pre-, co-, and post-treatment conditions, showing a noticeable synergy observed between TDZ and OFX (especially in pre-and co-treatment conditions). N=3.

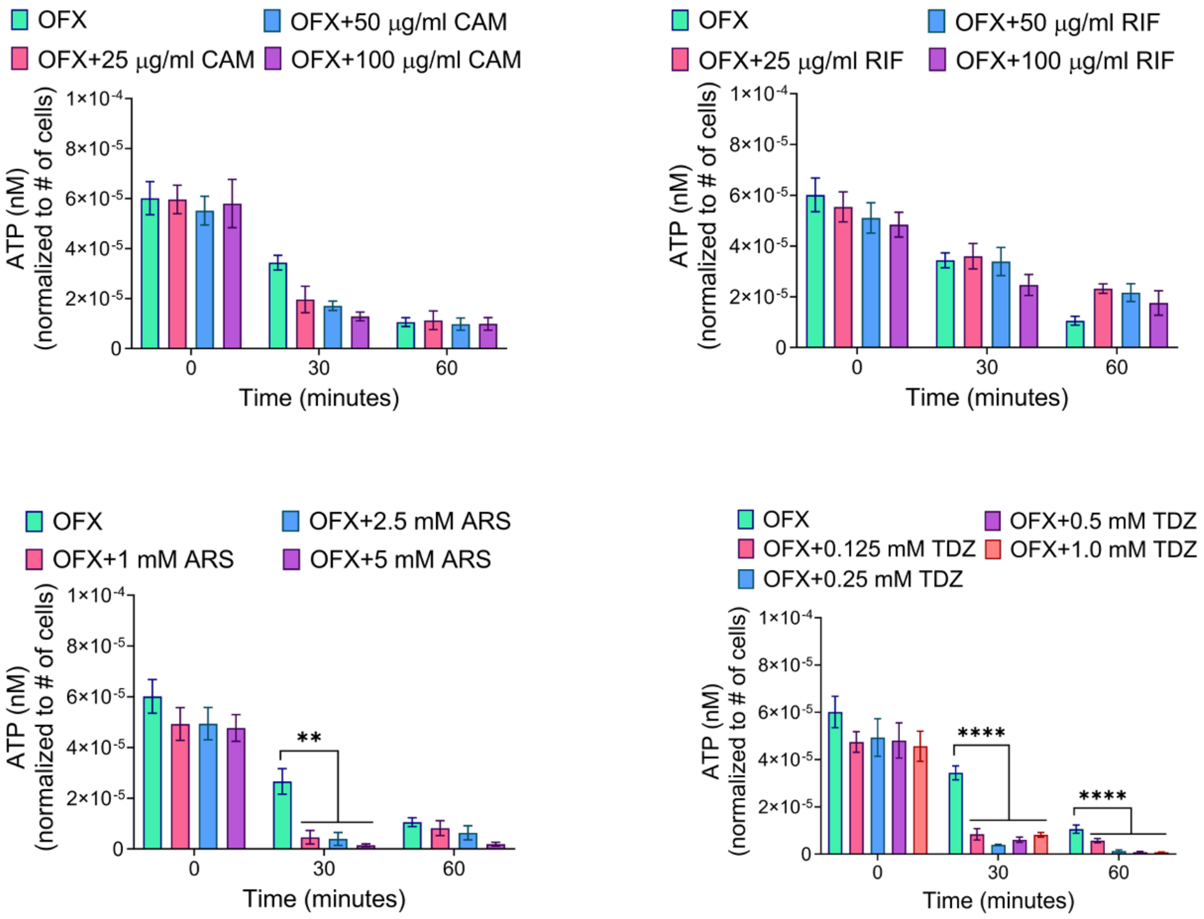

Supplementary Figure 3. The impact of metabolic inhibitors on cellular ATP levels. Metabolic inhibitors and OFX (5 μg/ml) were administered at t=5 hours. Culture samples were collected at the indicated time points during the treatments to quantify ATP levels. For pairwise comparisons, a one-way ANOVA with Dunnett's post-test was applied. The threshold values for statistical significance analysis were established as **P < 0.01, and ****P < 0.0001. The mean value ± standard deviation represents the data at each time point. ATP levels were normalized on a per-cell basis. N=3.

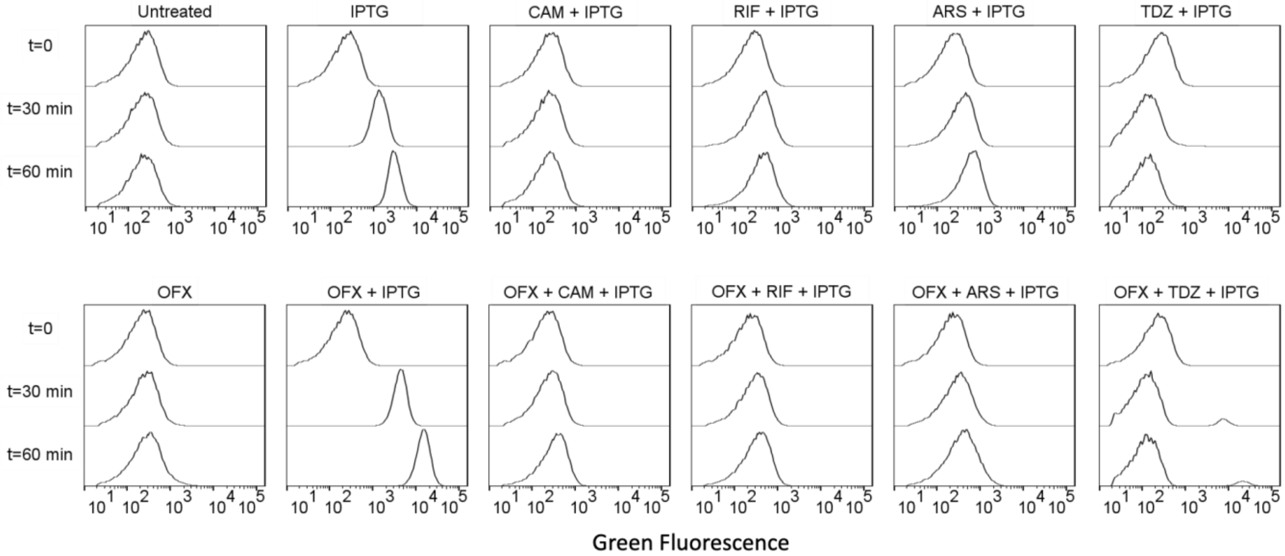

Supplementary Figure 4. The flow cytometry method used for quantifying GFP levels. Metabolic inhibitors (100 µg/ml CAM, 100 µg/ml RIF, 5 mM ARS, and 1 mM TDZ), and/or OFX (5 µg/ml), and/or 1 mM IPTG were introduced at t=5 hours. Culture samples were collected at specified intervals during the treatments to assess GFP levels. The figure depicts results from a representative biological replicate, with consistent outcomes observed across all replicates.

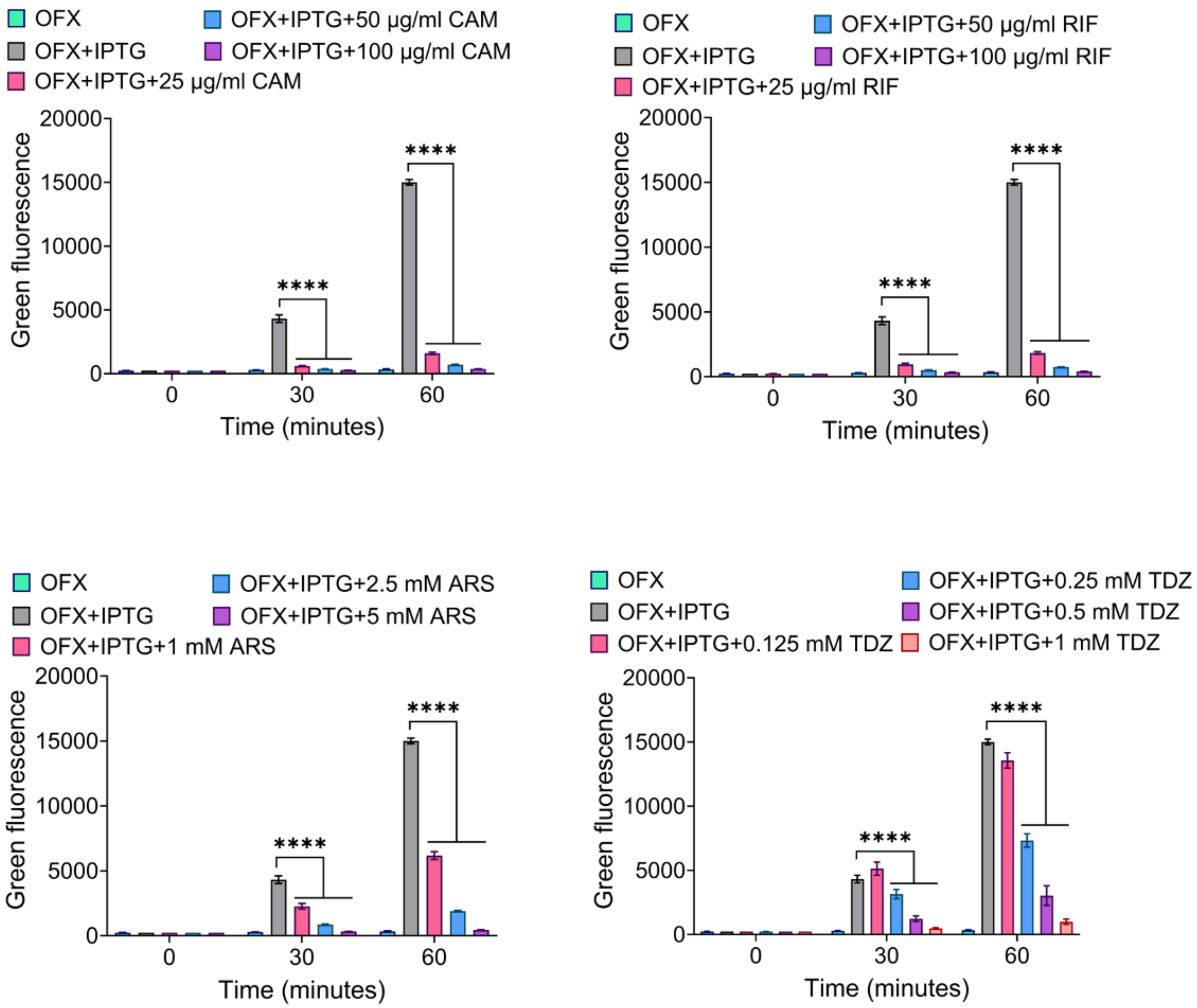

Supplementary Figure 5. The impact of metabolic inhibitors on transcription and translation activities. Metabolic inhibitors and OFX (5 μg/ml) together with 1 mM IPTG were administered at t=5 hours. Culture samples were collected at the indicated time points during the treatments to quantify GFP levels. For pairwise comparisons, a one-way ANOVA with Dunnett's post-test was applied. The threshold value for statistical significance analysis was established as ****$P < 0.0001$. The mean value ± standard deviation represents the data at each time point. N=3.

Supplementary Table 1. CFU and persister fractions for pre-, co-, and post-treatment conditions. Cells were exposed to OFX (5 µg/ml) at t=5 hours. The inhibitors were introduced at specified concentrations either 1 hour before OFX addition (pre-treatment), simultaneously with OFX addition (co-treatment), or 1 hour after OFX addition (post-treatment). CFU levels were assessed before and after 20-hour-OFX treatments; persister fractions represent the ratio of CFU levels after OFX treatment to those before treatment. Cell cultures had approximately 1-2 billion (10^9) CFU/ml before OFX treatment. N=3. Rep.: Replicate. ULD: Under limit of detection ($<10^{-9}$).

**Pre-treatment**	**Persister fraction**		
	**1st Rep.**	**2nd Rep.**	**3rd Rep.**
**OFX only**	7.7E-06	9.5E-06	2.8E-05
**OFX+25 µg/ml CAM**	2.2E-04	1.7E-04	2.3E-04
**OFX+50 µg/ml CAM**	1.4E-03	1.3E-03	1.2E-03
**OFX+100 µg/ml CAM**	4.1E-03	2.1E-03	2.3E-03
**OFX+25 µg/ml RIF**	1.6E-05	1.2E-05	1.7E-05
**OFX+50 µg/ml RIF**	2.4E-05	1.4E-05	3.8E-05
**OFX+100 µg/ml RIF**	2.5E-05	1.9E-05	2.5E-05
**OFX+1 mM Ars**	1.2E-03	4.8E-04	2.8E-03
**OFX+2.5 mM Ars**	1.6E-03	1.6E-03	1.2E-03
**OFX+5 mM Ars**	1.0E-03	9.1E-04	2.6E-03
**OFX+0.125 mM TDZ**	3.9E-07	1.6E-07	8.5E-07
**OFX+0.25 mM TDZ**	ULD	ULD	ULD
**OFX+0.5 mM TDZ**	ULD	ULD	ULD
**OFX+1 mM TDZ**	ULD	ULD	ULD

**Co-treatment**	**Persister fraction**		
	**1st Rep.**	**2nd Rep.**	**3rd Rep.**
**OFX only**	2.4E-06	3.5E-06	1.5E-06
**OFX+25 µg/ml CAM**	4.0E-03	6.1E-04	7.9E-04
**OFX+50 µg/ml CAM**	1.4E-03	8.7E-05	9.4E-04
**OFX+100 µg/ml CAM**	2.8E-03	7.4E-05	8.5E-04
**OFX+25 µg/ml RIF**	8.0E-06	2.6E-06	1.2E-06
**OFX+50 µg/ml RIF**	4.0E-05	2.6E-06	2.9E-06
**OFX+100 µg/ml RIF**	2.8E-04	9.6E-06	7.9E-05
**OFX+1 mM Ars**	1.2E-04	2.7E-04	1.4E-04
**OFX+2.5 mM Ars**	7.6E-05	8.7E-04	8.8E-05
**OFX+5 mM Ars**	1.5E-04	1.2E-03	8.2E-05
**OFX+0.125 mM TDZ**	1.6E-08	2.0E-08	4.4E-09
**OFX+0.25 mM TDZ**	ULD	ULD	ULD
**OFX+0.5 mM TDZ**	ULD	ULD	ULD
**OFX+1 mM TDZ**	ULD	ULD	ULD

**Post-treatment**	**Persister fraction**		
	**1st Rep.**	**2nd Rep.**	**3rd Rep.**
**OFX only**	1.5E-05	2.3E-05	6.3E-06
**OFX+25 µg/ml CAM**	3.0E-04	3.5E-04	1.5E-04
**OFX+50 µg/ml CAM**	2.9E-04	3.9E-03	2.6E-03
**OFX+100 µg/ml CAM**	2.4E-04	5.3E-03	2.4E-03
**OFX+25 µg/ml RIF**	2.2E-06	3.8E-05	1.6E-05
**OFX+50 µg/ml RIF**	2.4E-06	2.5E-05	2.2E-05
**OFX+100 µg/ml RIF**	3.1E-06	4.3E-05	2.8E-05
**OFX+1 mM Ars**	1.4E-03	2.0E-02	6.3E-03
**OFX+2.5 mM Ars**	2.1E-03	2.3E-02	1.9E-02
**OFX+5 mM Ars**	3.0E-03	1.0E-02	9.4E-03
**OFX+0.125 mM TDZ**	5.5E-06	5.4E-06	2.2E-06
**OFX+0.25 mM TDZ**	3.0E-06	3.1E-06	2.1E-06
**OFX+0.5 mM TDZ**	2.1E-06	3.3E-06	1.1E-06
**OFX+1 mM TDZ**	1.0E-06	1.6E-06	9.4E-07
