# Supplementary material for: Unveiling the critical roles of cellular metabolism suppression in antibiotic tolerance
Source: NPJ Antimicrob Resist. 2024 Jun 24;2:17. doi: 10.1038/s44259-024-00034-7 (PMC11721439; doi:10.1038/s44259-024-00034-7)
Supplement: Supplementary file 2 — Reporting Summary [file 44259_2024_34_MOESM2_ESM.pdf]

nature portfolio

Corresponding author(s): Mehmet A. Orman

Last updated by author(s): Mar 1, 2024

Reporting Summary

Nature Portfolio wishes to improve the reproducibility of the work that we publish. This form provides structure for consistency and transparency in reporting. For further information on Nature Portfolio policies, see our Editorial Policies and the Editorial Policy Checklist.

Statistics

For all statistical analyses, confirm that the following items are present in the figure legend, table legend, main text, or Methods section.

n/a	Confirmed	
☐	☒	The exact sample size (*n*) for each experimental group/condition, given as a discrete number and unit of measurement
☒	☐	A statement on whether measurements were taken from distinct samples or whether the same sample was measured repeatedly
☐	☒	The statistical test(s) used AND whether they are one- or two-sided<br>*Only common tests should be described solely by name; describe more complex techniques in the Methods section.*
☒	☐	A description of all covariates tested
☐	☒	A description of any assumptions or corrections, such as tests of normality and adjustment for multiple comparisons
☐	☒	A full description of the statistical parameters including central tendency (e.g. means) or other basic estimates (e.g. regression coefficient) AND variation (e.g. standard deviation) or associated estimates of uncertainty (e.g. confidence intervals)
☐	☒	For null hypothesis testing, the test statistic (e.g. *F*, *t*, *r*) with confidence intervals, effect sizes, degrees of freedom and *P* value noted<br>*Give P values as exact values whenever suitable.*
☒	☐	For Bayesian analysis, information on the choice of priors and Markov chain Monte Carlo settings
☒	☐	For hierarchical and complex designs, identification of the appropriate level for tests and full reporting of outcomes
☒	☐	Estimates of effect sizes (e.g. Cohen's *d*, Pearson's *r*), indicating how they were calculated

Our web collection on statistics for biologists contains articles on many of the points above.

Software and code

Policy information about availability of computer code

Data collection	Plate reader data were collected using SkanIt Software V 5.0. Flow cytometry data were colected using NovoExpress software.
Data analysis	Flow cytometry data analysis was performed using FlowJo V 10.10.0. Statistical analysis was performed with Prism V 9.3.0. MATLAB (V R2020b) was used.

For manuscripts utilizing custom algorithms or software that are central to the research but not yet described in published literature, software must be made available to editors and reviewers. We strongly encourage code deposition in a community repository (e.g. GitHub). See the Nature Portfolio guidelines for submitting code & software for further information.

Data

Policy information about availability of data

All manuscripts must include a data availability statement. This statement should provide the following information, where applicable:

- Accession codes, unique identifiers, or web links for publicly available datasets
- A description of any restrictions on data availability
- For clinical datasets or third party data, please ensure that the statement adheres to our policy

All data in this manuscript is available in either the Main Text or Supplementary File. Additionally, raw data will be promptly provided upon request.

Research involving human participants, their data, or biological material

Policy information about studies with human participants or human data. See also policy information about sex, gender (identity/presentation), and sexual orientation and race, ethnicity and racism.

Reporting on sex and gender	Not applicable in this study.
Reporting on race, ethnicity, or other socially relevant groupings	Not applicable in this study.
Population characteristics	Not applicable in this study.
Recruitment	Not applicable in this study.
Ethics oversight	Not applicable in this study.

Note that full information on the approval of the study protocol must also be provided in the manuscript.

Field-specific reporting

Please select the one below that is the best fit for your research. If you are not sure, read the appropriate sections before making your selection.

☒ Life sciences ☐ Behavioural & social sciences ☐ Ecological, evolutionary & environmental sciences

For a reference copy of the document with all sections, see nature.com/documents/nr-reporting-summary-flat.pdf

Life sciences study design

All studies must disclose on these points even when the disclosure is negative.

Sample size	For flow cytometry analysis, at least 30,000 events (cells) were analyzed.
Data exclusions	No data were excluded from any analysis.
Replication	A minimum of three independent biological replicates were performed for all experiments. The number of biological replicates was provided in the figure legends. The data points representing the biological replicates were also shown on the panels.
Randomization	Not applicable in this study.
Blinding	Not applicable in this study.

Reporting for specific materials, systems and methods

We require information from authors about some types of materials, experimental systems and methods used in many studies. Here, indicate whether each material, system or method listed is relevant to your study. If you are not sure if a list item applies to your research, read the appropriate section before selecting a response.

Materials & experimental systems

n/a	Involved in the study	
☒	☐	Antibodies
☒	☐	Eukaryotic cell lines
☒	☐	Palaeontology and archaeology
☒	☐	Animals and other organisms
☒	☐	Clinical data
☒	☐	Dual use research of concern
☒	☐	Plants

Methods

n/a	Involved in the study	
☒	☐	ChIP-seq
☐	☒	Flow cytometry
☒	☐	MRI-based neuroimaging

Plants

Seed stocks	Not applicable in this study.
Novel plant genotypes	Not applicable in this study.
Authentication	Not applicable in this study.

Flow Cytometry

Plots

Confirm that:

☒ The axis labels state the marker and fluorochrome used (e.g. CD4-FITC).

☒ The axis scales are clearly visible. Include numbers along axes only for bottom left plot of group (a 'group' is an analysis of identical markers).

☒ All plots are contour plots with outliers or pseudocolor plots.

☒ A numerical value for number of cells or percentage (with statistics) is provided.

Methodology

Sample preparation	Overnight cultures were diluted in fresh LB medium, and treated with antibiotics/chemicals at the indicated growth phase. Treated cells were then collected at indicated time points and analyzed with the flow cytometer.
Instrument	NovoCyte Flow Cytometer, NovoCyte 3000RYB, ACEA Biosciences Inc., San Diego, CA, United States
Software	NovoExpress Software was used to collect the data. FlowJo software was used to analyze the data.
Cell population abundance	Cells were diluted in PBS to reach a desired cell density for the flow analysis (10^6–10^7 cells/ml) for each flow sample.
Gating strategy	To eliminate the instrument noise from the flow cytometry data, PBS solution without bacterial cells was used.

☒ Tick this box to confirm that a figure exemplifying the gating strategy is provided in the Supplementary Information.
